# Transcriptome Analyses Reveal Systematic Molecular Pathology After Optic Nerve Crush

**DOI:** 10.3389/fncel.2021.800154

**Published:** 2022-01-10

**Authors:** Yuan-Bo Pan, Yiyu Sun, Hong-Jiang Li, Lai-Yang Zhou, Jianmin Zhang, Dong-Fu Feng

**Affiliations:** ^1^Department of Neurosurgery, Southern Medical University Affiliated Fengxian Hospital, Shanghai, China; ^2^Department of Neurosurgery, Second Affiliated Hospital, School of Medicine, Zhejiang University, Hangzhou, China; ^3^Department of Neurosurgery, The First Affiliated Hospital of Zhengzhou University, Zhengzhou, China; ^4^Brain Research Institute, Zhejiang University, Hangzhou, China; ^5^Collaborative Innovation Center for Brain Science, Zhejiang University, Hangzhou, China

**Keywords:** optic nerve crush, WGCNA, transcriptome, microglia, immune response

## Abstract

The function of glial cells in axonal regeneration after injury has been the subject of controversy in recent years. Thus, deeper insight into glial cells is urgently needed. Many studies on glial cells have elucidated the mechanisms of a certain gene or cell type in axon regeneration. However, studies that manipulate a single variable may overlook other changes. Here, we performed a series of comprehensive transcriptome analyses of the optic nerve head over a period of 90 days after optic nerve crush (ONC), showing systematic molecular changes in the optic nerve head (ONH). Furthermore, using weighted gene coexpression network analysis (WGCNA), we established gene module programs corresponding to various pathological events at different times post-ONC and found hub genes that may be potential therapeutic targets. In addition, we analyzed the changes in different glial cells based on their subtype markers. We revealed that the transition trend of different glial cells depended on the time course, which provides clues for modulating glial function in further research.

## Introduction

Axons of the adult mammalian central nervous system (CNS) fail to regenerate after injury mainly due to the following: 1. The poor intrinsic regenerative capacity of neurons. 2. Multiple inhibitory factors in the surrounding glial environment. In recent decades, although long distance and sustained axon regeneration have been realized by manipulating certain genes in neurons (Park et al., 2008; Moore et al., 2009; Sun et al., 2011), there are still many unsolved problems with this process, such as misguidance, poor conduction and non-functional synapses, which are closely related to glial cell function (Pernet et al., 2013; Bei et al., 2016). In the process of regrowth, regenerating axons bind to contact astrocytic scars or myelin debris, which are traditionally believed to be inhibitory factors. However, this view was challenged by recent evidence that astrocyte scar formation aids CNS axon regeneration (Anderson et al., 2016). In addition, a recent study proved that microglia are irrelevant for neuronal degeneration and axon regeneration after acute injury (Hilla et al., 2017). All these findings indicate that the function of glial cells changes dynamically during injury progression, and we should reexamine their roles in this pathological processes.

Axons of retinal ganglion cells converge at the fovea and pass through the eyeball to form the optic nerve. Then, unmyelinated axons at the optic nerve head (ONH) region continue as a series of myelinated bundles toward the brain (Williams et al., 2020). This structure is similar to that of white matter in the brain parenchyma and spinal cord. The interstitium of the optic nerve is filled with glial cells, including astrocytes, oligodendrocytes and microglia. In addition, the dura that covers the optic nerve is a direct continuation of the meningea (Shoja and Oyesiku, 2014). Thus, essentially, the optic nerve is a white matter tract of the brain that connects the eye with the brain and is considered to be part of the CNS. Due to its relatively simple anatomical structure, optic nerve injury has become a widely used model to study the interactions between glial cells and axons without considering their complex role in maintaining synapse functionality, such as that in the brain. Notably, the characteristics of the optic nerve regenerative response are shared by other CNS tissues, including the brain and spinal cord.

Injury in axons triggers a series of cellular and molecular responses in neighboring glial cells, including demyelination, astrocyte activation and microglial infiltration. These pathological alterations are continuous, dynamically changing and mutually influence each other. Various glial cells in the optic nerve respond inconsistently to injury. Even the same type of cell presents different morphological changes and gene expression at different periods, thus resulting in different roles at the corresponding time points (Tedeschi and Bradke, 2017; Yazdankhah et al., 2021). Studies have proven that such alterations have an important impact on regenerative outcomes (Anderson et al., 2016; Li et al., 2020). Although few studies have attempted to reveal the gene expression pattern during the pathogenic progress of injury (Agudo et al., 2008; Qu and Jakobs, 2013; Sharma et al., 2014), limited by the data processing capability, most studies have only examined simple gene expression and may overlook many details.

In this paper, using a powerful bioinformatics tool known as weighted gene coexpression network analysis (WGCNA), we analyzed the transcriptome data of the ONH after optic nerve crush and established gene modules and programs representing various events at different times after optic nerve crush (ONC). In addition, we revealed potential key regulatory factors by hub gene network analyses. Compared with a previous study, we combined isolated or incoherent methods into a framework and provided a more holistic and objective view of the pathological process in the optic nerve head.

## Methods

### Dataset Preparation

A total of 55 optic nerve head (ONH) samples of C57BL/6 mice were included in this study. These samples were divided into two batches. The first batch of samples included 5 naïve control ONHs (0 d), 5 ipsilateral ONHs 1 d after optic nerve crush (ONC), 5 contralateral ONHs 1 d after ONC, 5 ipsilateral ONHs 3 d after ONC, 5 contralateral ONHs 3 d after ONC, 5 ipsilateral ONHs 7 d after ONC, 5 contralateral ONHs 7 d after ONC, 5 ipsilateral ONHs 21 d after ONC, and 5 contralateral ONHs 21 d after ONC. The second batch of samples contained 5 ipsilateral ONHs 90 d after ONC and 5 contralateral ONHs 90 d after ONC. The method of optic nerve crush was performed according to previously reported literature (Qu and Jakobs, 2013). The raw gene expression data of the GSE40857 dataset were accessed from the Gene Expression Omnibus database. The corresponding sample traits were also acquired. The gene expression profiles of optic nerve heads based on Affymetrix Mouse Genome 430A 2.0 Array were used for analysis.

### Data Processing

Using the robust multiarray average (RMA) algorithm in the Affy package, we processed the raw probe-level data in the CEL file with background correction, quartile data normalization and conversion into expression measures (Irizarry et al., 2003). For genes that correspond to multiple probes, we used the average probe value as the expression value. Missing data in gene expression matrices were imputed with the k-nearest neighbor (KNN) approach (*k* = 10) (Garcia-Laencina et al., 2015).

### Principal Component Analysis

Principal component analysis (PCA) was performed on the optic nerve head samples using all genes expressed in more than two samples. With R language (v. 3.4.3) and visualization using the “ggplot2” package, PCA was performed to determine the principal components and to identify the differences in gene expression patterns among these optic nerve head samples. Each sample was differentially colored and shaped according to the optic nerve crush and the time point of the crush.

### Differential Expression Analysis

We used the R package “limma” to identify differentially expressed genes (DEGs) between the contralateral control samples and the optic nerve-crushed samples at different time points (Ritchie et al., 2015). An adjusted *P*-value < 0.05 and log|foldchange| (log|FC|) > 0.5 were chosen to detect statistically significant DEGs.

### Enrichment Analyses

Gene Ontology (GO) enrichment analysis was performed using the DAVID platform (DAVID 6.8, https://david.ncifcrf.gov/) (Huang Da et al., 2009). Genes with MM > 0.6 within each module were utilized during GO enrichment analyses. GO terms with Benjamini corrected *P*-values < 0.01 are shown in Supplementary Table 1. With the CTen database0 (http://sbi.jp/influenza-x/cten/), cell enrichment analysis was performed on genes with MM > 0.6 within each module. The enrichment score (–log_10_ Benjamini-Hochberg adjusted *P*-values) of each module was calculated. The cell type terms with the highest enrichment scores are shown in **Figure 3**. All the cell type terms and their enrichment scores are shown in Supplementary Table 2.

### Construction of Coexpression Networks

A weighted gene coexpression network was constructed to identify gene modules associated with the expression patterns of ONHs after crush following a previously described method (Pan et al., 2020). The coexpression network was constructed by the R package “WGCNA” (Langfelder and Horvath, 2008). In brief, the correlation network was first constructed by creating a matrix of Pearson's correlation between all pairwise genes. Next, with a power (β) of 18, the obtained correlation matrix was changed into a weighted adjacency matrix (Zhang and Horvath, 2005). Then, a measure of network interconnectedness, topological overlap matrix (TOM), was calculated (Zhang and Horvath, 2005). Based on the TOM-based dissimilarity measure, we used average linkage hierarchical clustering to classify genes with similar expression patterns into the same modules (Langfelder and Horvath, 2008). The minimum size of each module was 30 genes. Using a dynamic tree-cutting algorithm, we identified the preliminary modules. Then, the final module was determined via a merging threshold function at 0.25 (Zhang and Horvath, 2005).

### Calculating Module Eigengenes, Module Membership and Intramodular Connectivity

The first principal component of each module was regarded as the module eigengene (ME). The Pearson correlation between that gene and the ME in a given module was regarded as module membership (MM) of this gene. The gene of a given module with a higher MM plays a more important role in the module. The genes with MM > 0.6 were chosen for subsequent analyses. The intramodular connectivity of one gene in a given module was defined as the sum of the correlation coefficients with other nodes in this module. The genes with higher intramodular connectivity also play crucial roles in the module.

### Visualization of Key Networks

For each module, Cytoscape 3.8.0 was used to visualize the top 250 connections (based on topological overlap). The M1 and M9 modules contained fewer than 250 connections; thus, the total connections of the two modules were visualized. The hub genes in each module were marked as large-sized nodes.

### Statistical Analysis

GraphPad Prism (version 6.0, GraphPad Software, San Diego, CA, USA) and R language (3.4.0) were used for statistical analysis. Significant differences were determined by Student's *t*-test for two-group comparisons. The results are presented as means ± SD. A *P*-value < 0.05 was considered statistically significant.

## Results

### Temporal Changes Following ONC Revealed by PCA and Sample Clustering

**Figure 1:**
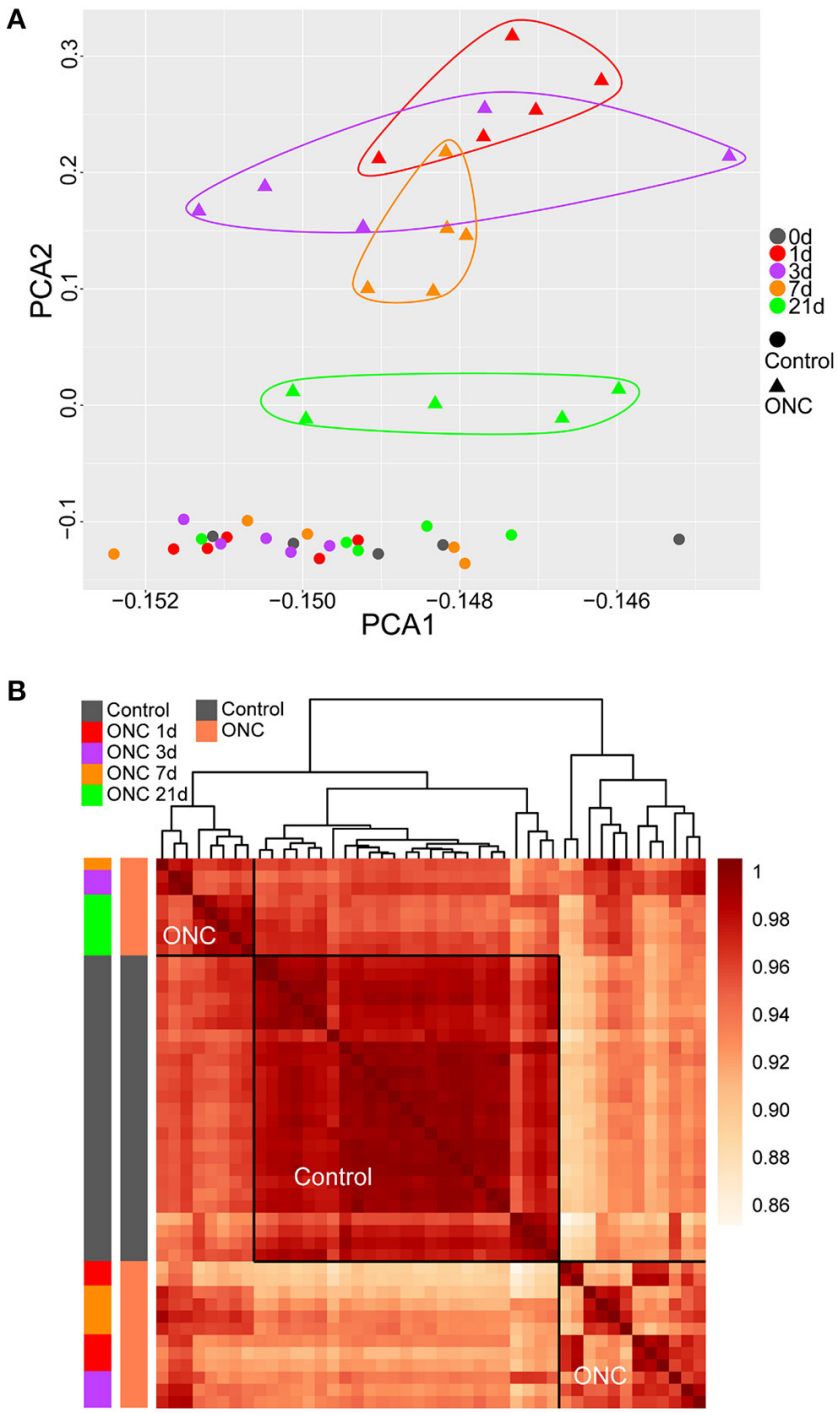
PCA and sample clustering revealed temporal changes following ONC. **(A)** PCA of 45 ONH samples. The time points following ONC are labeled with different colors. Uninjured control ONHs are labeled with circles. The injured ONHs are labeled with triangles. **(B)** Heatmap of unbiased hierarchical clustering of pairwise correlations among all 45 samples. The dark red in the heatmap represents Pearson's correlation coefficient of transcriptomes among pairs of samples in all combinations. The traits of all samples are labeled with different colors. The horizontal and vertical frames are symmetrical. All the uninjured control samples were clustered together. ONH, optic nerve head; PCA, principal component analysis; ONC, optic nerve crush.

First, all gene profiles of the complete dataset (55 ONHs) were used to perform PCA. The two- and three-dimensional PCAs showed that the contralateral ONHs 90 d post-ONC (no surgery) were different from the naïve control samples and the contralateral ONHs at other time points (Supplementary Figures 1A,B), which confirmed that there was a batch effect between the ONHs at 90 d and the ONHs at other time points (0/1/3/7/21 d), as mentioned in the Methods section. Hierarchical clustering of the complete dataset based on all gene profiles also showed the batch effect (Supplementary Figure 1C). Thus, the first batch of ONHs (0/1/3/7/21 d) was used to perform sample clustering. Two-dimensional PCA of all gene profiles of the first batch groups revealed the transcriptome differences caused by time differences (Figure 1A). The small transcriptome differences among the whole uninjured control ONHs also suggested good sample uniformity. The differences in expression patterns between the ONHs 1 d post-ONC (red triangles) and the normal ONHs (rounds) were the largest (Figure 1A), suggesting that the ONHs 1 d post-ONC underwent the most dramatic biological changes. The differences in expression patterns between the crushed ONHs and the uninjured control ONHs gradually decreased as the recovery time after ONC increased (Figure 1A). The ONHs 21 d post-ONC (green triangles) were closer to the uninjured ONHs than to the other ONHs post-ONC (triangles with other colors), suggesting that the ONHs 21 d post-ONC would recover similar to normal tissues (Figure 1A). However, the expression patterns of the ONHs 21 d post-ONC were still different from those of the uninjured control ONHs, indicating that the damage caused to optic nerves had not fully recovered after 3 weeks (Figure 1A).

Based on Pearson's correlation coefficient of transcriptomes among pairs of samples in all combinations, unbiased hierarchical clustering of the 45 ONHs divided the samples into three major groups (Figure 1B). In Figure 1B, the darker the color, the higher the correlation was. All uninjured control ONHs formed one cluster, and the other ONHs at different time points after ONC formed two clusters. The ONHs 21 d post-ONC and some ONHs 3 d/7 d post-ONC clustered together and were more closely correlated with the uninjured control ONHs than with the ONHs 1 d post-ONC, suggesting more dramatic tissue crushing and more significant changes in expression patterns within ONHs, whereas injured tissues were gradually repaired 3 days after ONC (Figure 1B). The ONHs 21 d post-ONC correlated better with the uninjured control samples than the ONHs 1 d post-ONC, indicating that the expression patterns of the ONHs 21 d post-ONC were more similar to those of the normal samples (Figure 1B). Based on the whole gene profile of each ONH, hierarchical clustering of the 45 ONHs also showed significant transcriptome differences caused by time differences (Supplementary Figure 2).

### Temporal Expression of Genes Following ONC

**Figure 2:**
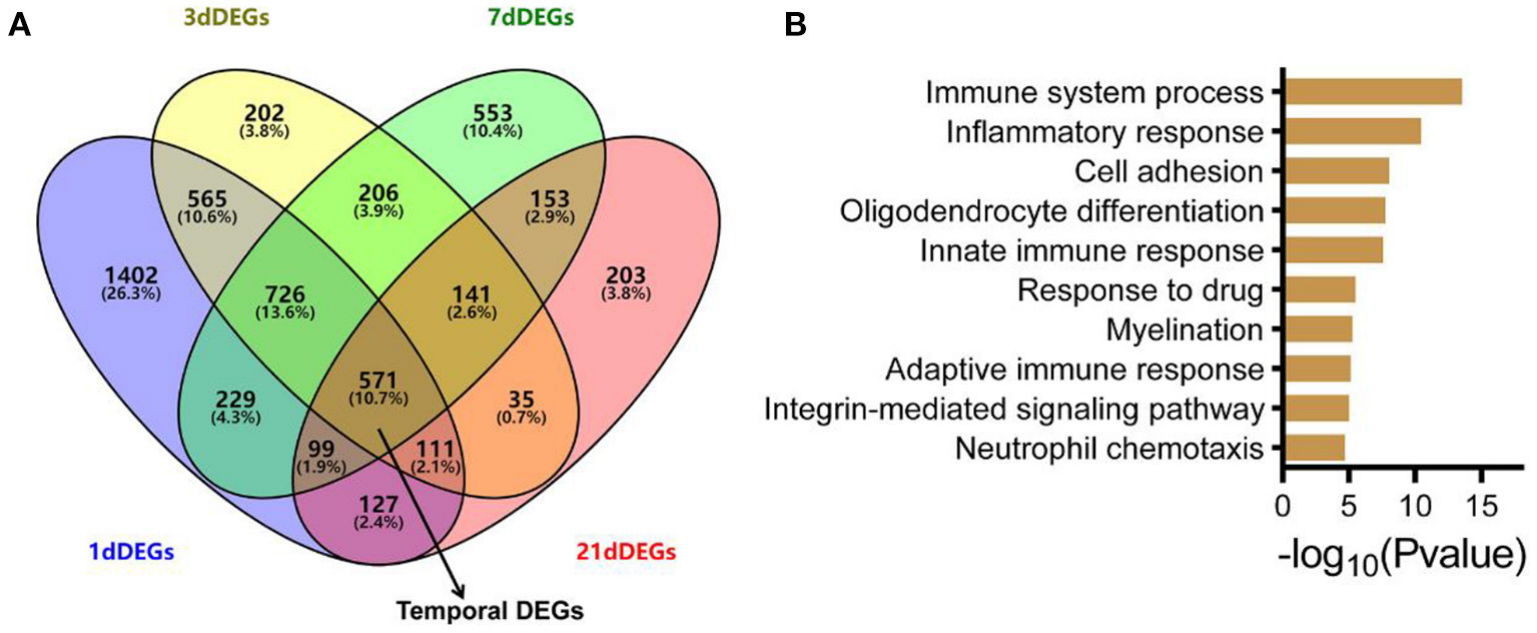
ONC-induced temporal DEGs and the involved biological processes. **(A)** Venn plot of DEGs 1/3/7/21 d post-ONC. The temporal DEGs and overlap of the four DEGs are indicated by black arrows. **(B)** GO enrichment analysis for temporal DEGs showed the top 10 most enriched biological processes. GO, Gene Ontology; DEG, differentially expressed gene; ONC, optic nerve crush.

To identify differentially expressed genes (DEGs) at each time point, we used the R package limma (Ritchie et al., 2015). Under different threshold conditions (logFC), the numbers of DEGs at each time point after ONC are shown (Supplementary Figure 3A). The lists of the DEGs at each time point after ONC are also shown in Supplementary Table 3. Moreover, volcano plots of the DEGs are shown (Supplementary Figures 3B–E). There were 3830 and 2557 DEGs in the ONHs 1 d post-ONC and the ONHs 3 d post-ONC relative to their contralateral ONHs (Figure 2A). The number of DEGs between the ONHs 7 d post-ONC and the contralateral ONHs was 2,678, and the number of DEGs between the ONHs 21 d post-ONC and the contralateral ONHs was 1,440 (Figure 2A). These results are consistent with the PCA, which suggests that time had a significant influence on gene expression. Temporal DEGs were defined as genes that changed continuously at different time points. Thus, the number of temporal DEGs following ONC was 571 (Figure 2A). Functional enrichment analysis (GO biological process) of the temporal genes showed that these genes are highly associated with categories related to immune system processes, inflammatory responses, cell adhesion, oligodendrocyte differentiation and myelination (Figure 2B). These analyses indicated that we could use these data to explore molecular correlates of injury repair events following ONC.

### Using WGCNA to Establish Gene Modules Underlying Pathological Events Post-ONC

**Figure 3:**
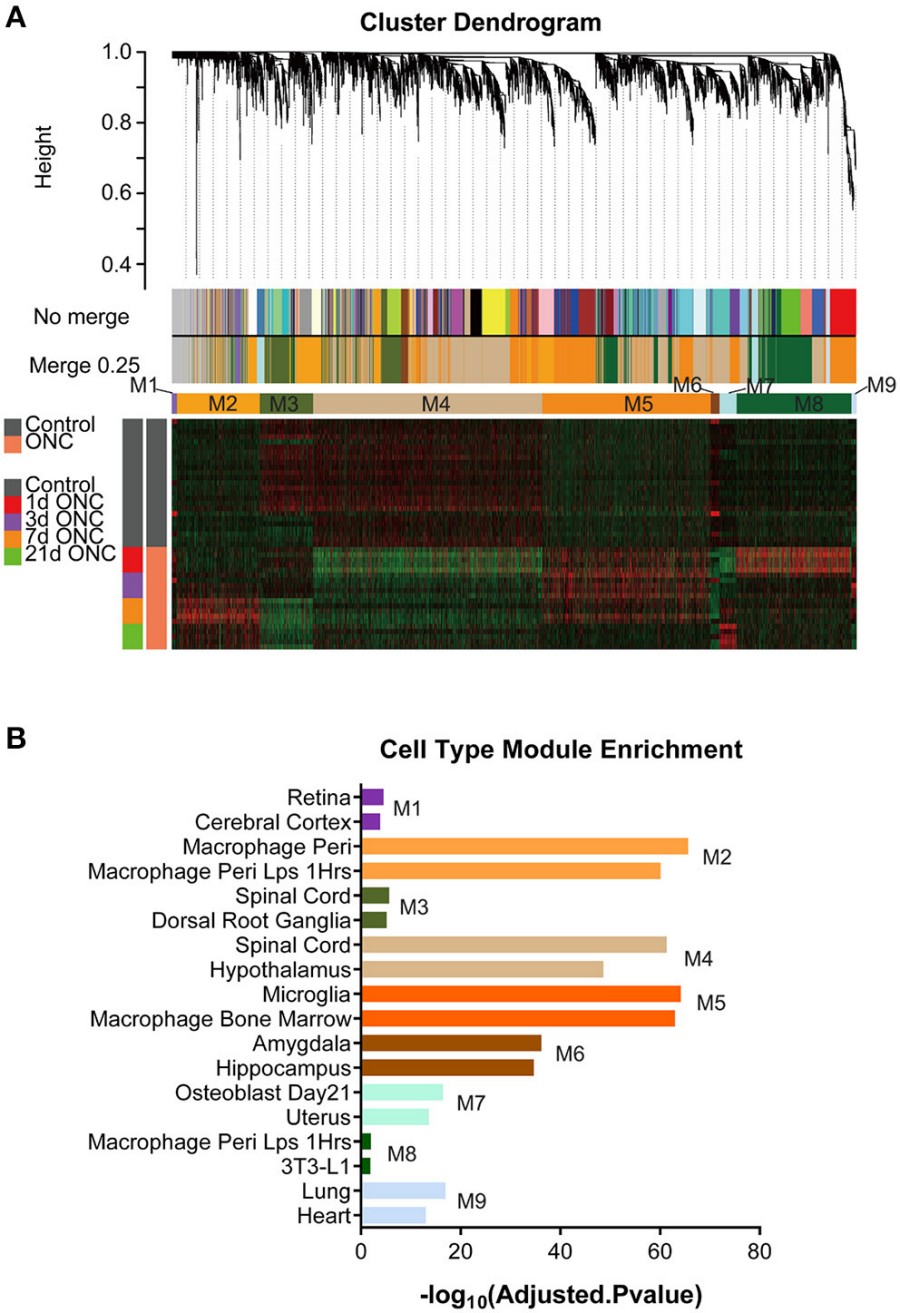
WGCNA of all 45 ONH samples identified gene modules. **(A)** With 5,323 time-course-related genes, a hierarchical cluster dendrogram of 45 ONH samples identified coexpression modules. Modules corresponding to the branches are labeled with different colors indicated by the color bands below the tree. Nine gene modules were generated after 0.25 threshold merging. **(B)** Cell type enrichment analyses for the 9 modules. The two most enriched cell type terms for each module are shown. ONH, optic nerve head.

**Figure 4:**
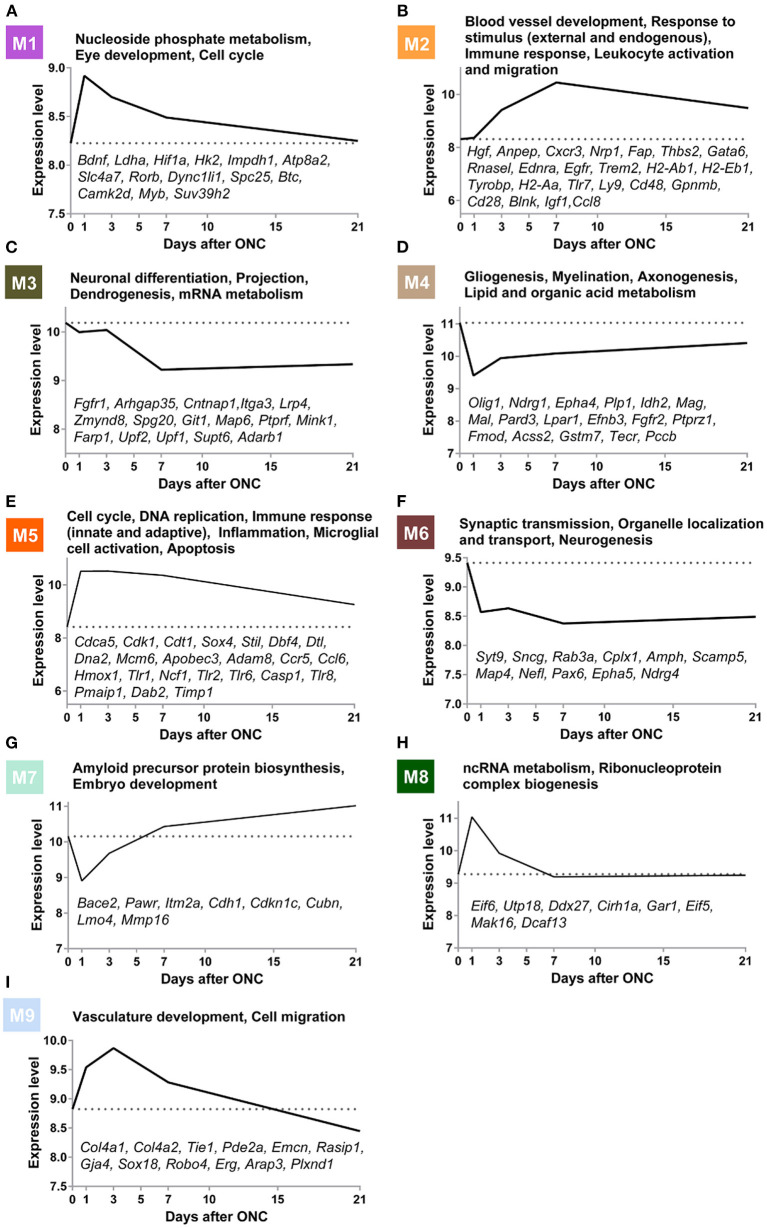
Gene modules underlying biological and pathological events at different time points post-ONC. **(A–I)** GO terms associated with the M1–M9 modules are shown. The time-course expression curves of the 9 modules based on the average expression levels of the top 30 genes with the highest MM are shown. The gene symbols of crucial genes in each module are shown. GO, Gene Ontology; ONC, optic nerve crush.

To further explore the biological and pathological events occurring following ONC, we subjected 45 ONHs (all uninjured control ONHs and injured ONHs 1/3/7/21 d post-ONC) to WGCNA, with an emphasis on genes that underwent crush-induced changes (5,323 genes were included), which could digitally represent biological and pathological cellular events in the ONH following ONC. WGCNA identified 9 gene modules/clusters (designated M1–M9) of coexpressed genes (Figures 3A,B, 4). Cell type enrichment for each module was performed. The two most enriched cell type terms for each module are shown (Figure 3B, Supplementary Figures 4, 5). For example, the terms “retina” and “cerebral cortex” were enriched in the M1 module. The terms “macrophage peri” and “macrophage peri LPS 1 Hrs” were significantly enriched in the M2 module. “Microglia” and “macrophage bone marrow” were significantly enriched in the M5 module.

Gene Ontology (GO) analysis of the 9 modules revealed several crucial biological processes that occurred in a temporally specific manner following ONC (Figure 4). In addition, the time-course expression curves in these 9 modules based on the average expression levels of the top 30 genes with the highest MM were plotted (Figure 4). GO analysis of the M6 module revealed that synaptic transmission-related genes (Syt9, Sncg, Rab3a, Cplx1, Amph, Scamp5), neurogenesis-related genes (Epha5, Map4, Ndrg4), and organelle localization and transport-related genes (Map4, Nefl, Pax6, Syt9) showed significantly downregulated expression immediately after injury, and the expression remained low even at Day 21 post-injury, indicative of long-term impairment. However, the expression of genes (M3) involved in neuronal differentiation (Fgfr1, Arhgap35, Cntnap1, Itga3), projection (Lrp4, Zmynd8, Spg20, Git1), dendrogenesis (Map6, Ptprf, Mink1, Farp1), and mRNA metabolism (Upf2, Upf1, Supt6, Adarb1) decreased slowly after injury and remained low for at least 21 d post-injury. In M1, a module related to nucleoside phosphate metabolism (Bdnf, Ldha, Hif1a, Hk2, and Impdh1), eye development (Atp8a2, Slc4a7, and Rorb), and the cell cycle (Dync1li1, Spc25, Btc, Camk2d, Myb, and Suv39h2), gene expression increased immediately after injury but then reversed relatively quickly during the first 7 d post-ONC and continued to slowly decrease, reaching almost normal (uninjured) levels by Day 21. The expression of genes (M4) involved in gliogenesis (Olig1, Ndrg1, Epha4, Plp1, Idh2), myelination (Mag, Mal, Pard3, Lpar1), axonogenesis (Efnb3, Fgfr2, Ptprz1, Fmod), and lipid and organic acid metabolism (Acss2, Gstm7, Tecr, Pccb) decreased quickly 1 d post-injury but then rose slightly at Day 3 post-ONC and thereafter continued to slowly increase to close to normal (uninjured) levels. In contrast, the expression of genes (M5) involved in the cell cycle (Cdca5, Cdk1, Cdt1, Sox4, Stil), DNA replication (Dbf4, Dtl, Dna2, Mcm6), immune response (Apobec3, Adam8, Ccr5, Ccl6), inflammation (Hmox1, Tlr1, Ncf1), microglial cell activation (Tlr1, Tlr2, Tlr6, Casp1, Tlr8), and apoptosis (Pmaip1, Dab2, Timp1) rose quickly after injury and then continued to gradually revert back to close to normal (uninjured) levels. The expression of genes (M2) involved in blood vessel development (Hgf, Anpep, Cxcr3, Nrp1, Fap, Thbs2, and Gata6), response to external and endogenous stimulus (Rnasel, Ednra, Egfr, and Trem2), immune response (H2-Ab1, H2-Eb1, Tyrobp, H2-Aa, Tlr7, and Ly9), and leukocyte activation and migration (Cd48, Gpnmb, Cd28, Blnk, Igf1, and Ccl8) rose slowly during the first 7 d post-ONC and then gradually reverted back to close to normal (uninjured) levels. In M8, a module related to ncRNA metabolism (Eif6, Utp18, Ddx27, Cirh1a) and ribonucleoprotein complex biogenesis (Gar1, Eif5, Mak16, Dcaf13), the expression rose quickly at Day 1 post-ONC and then reversed to normal (uninjured) levels relatively quickly by Day 7 post-injury and remained normal at Day 21 post-injury. In M7, a module related to amyloid precursor protein biosynthesis (Bace2, Pawr, Itm2a) and embryo development (Cdh1, Cdkn1c, Cubn, Lmo4, Mmp16), expression dynamically decreased at Day 1 post-ONC and then rose above normal (uninjured) levels quickly by Day 7 post-injury and thereafter increased slowly over the 21 d period. In M9, a module related to vasculature development (Col4a2, Col4a2, Tie1, Pde2a, Emcn, Rasip1, Gja4, Sox18) and cell migration (Robo4, Erg, Arap3, Plxnd1) was dynamically upregulated during the first 3 d and continued to slowly decrease below normal (uninjured) levels by Day 21 post-injury. Taken together, these results suggest that WGCNA can provide a digitized image of the biological/pathological events after ONC.

### Visualized Networks of Core Genes in Modules

**Figure 5:**
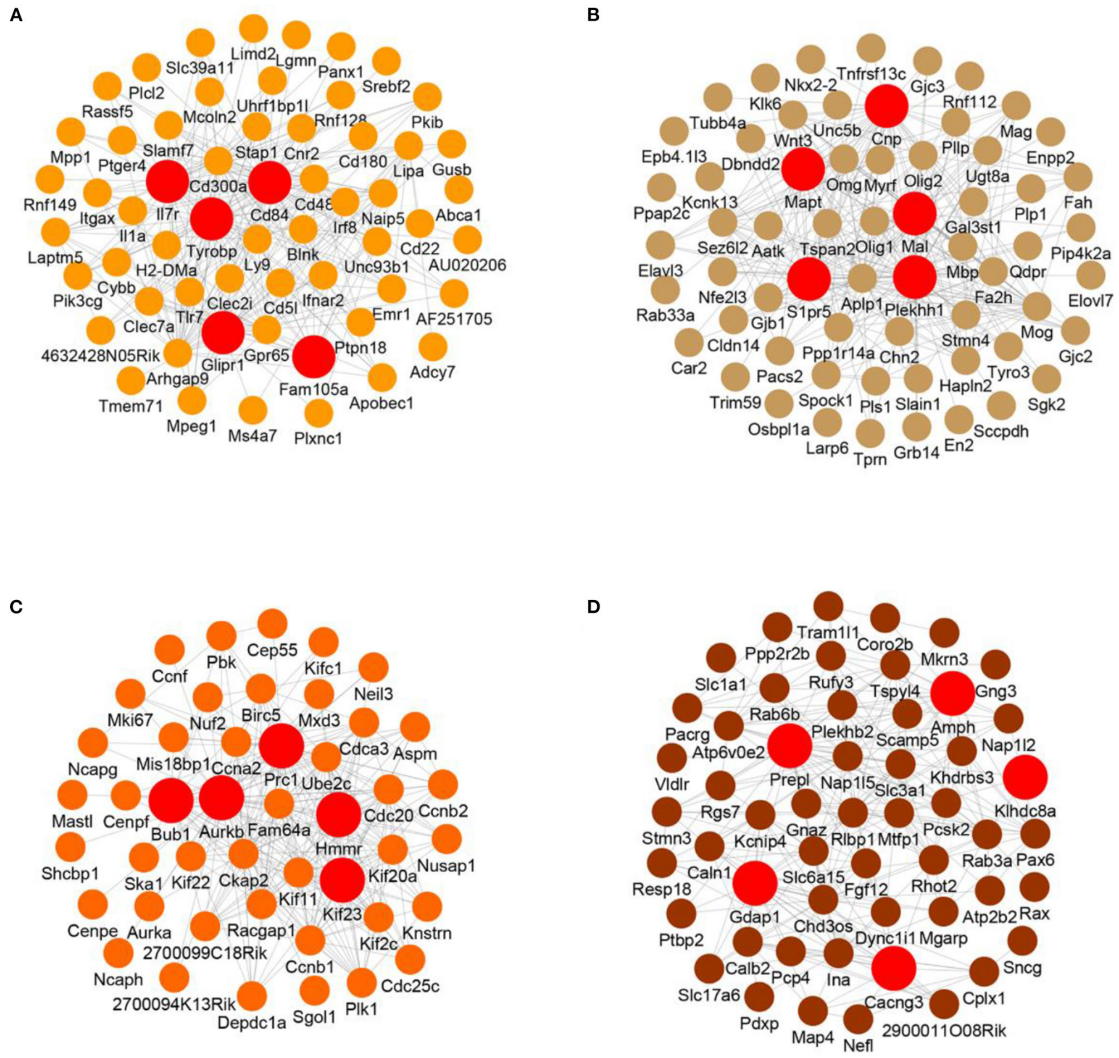
Visualized network of each module. **(A–D)** Visualized networks based on the top 250 gene connections of the M2 **(A)**, M4 **(B)**, M5 **(C)**, and M6 **(D)** modules. The top 5 hub genes based on degree in each module are shown.

To further analyze the interaction of genes in the modules, we visualized the top 250 connections in each module as networks (Figures 5A–D and Supplementary Figures 6A–E). Moreover, the hub genes in each module were identified according to the numbers of their neighbors. The hub genes in the M2 module (blood vessel development and immune response) were Il7r, Cd84, Tyrobp, Glipr1 and Fam105a (Figure 5A). In the gliogenesis-related M4 module, the hub genes were Mal, Cnp, Mapt, S1pr5 and Plekhh1 (Figure 5B). In the M5 module (cell cycle and immune response), the hub genes were Prc1, Ube2c, Bub1, Aurkb and Kif23 (Figure 5C). The hub genes in the M6 module (synaptic transmission and neurogenesis) were Amph, Prepl, Klhdc8a, Gdap1 and Cacng3 (Figure 5D). The hub genes in other modules are also shown (Supplementary Figures 6A–E).

### Expression Pattern of ONHs 90 d Post-ONC

**Figure 6:**
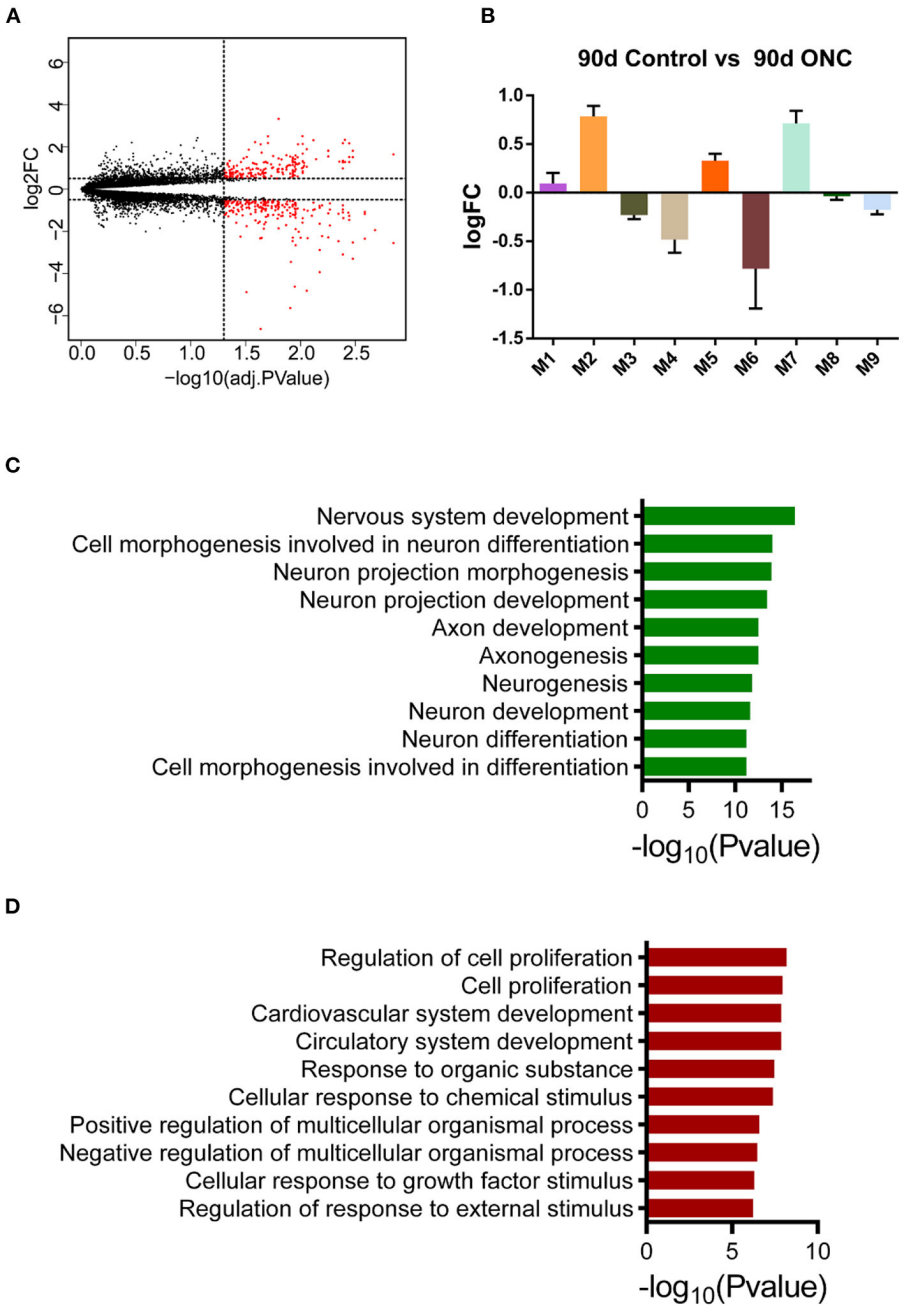
Expression pattern of ONHs 90 d post-ONC. **(A)** Volcano plot of DEGs in ONHs 90 d post-ONC compared with uninjured control ONHs. Red points represent DEGs. **(B)** The changes in the average expression levels of the top 30 genes with the highest MM in ONHs 90 d post-ONC. **(C,D)**. GO enrichment analyses of genes with downregulated **(C)** and upregulated **(D)** expression showed the top 10 enriched biological processes. DEG, differentially expressed gene; ONC, optic nerve crush; ONH, optic nerve head.

To further evaluate the long-term expression pattern of injured ONHs, we performed differential expression analysis for ONHs 90 d post-ONC. There were 443 DEGs between the ONHs 90 d post-ONC and the contralateral ONHs, including 203 genes with upregulated and 240 with downregulated expression (Figure 6A). In addition, we wondered whether the genes of these modules had changed 90 days following ONC. The average expression levels of the top 30 genes with the highest MM in these 9 modules are shown (Figure 6B), and we found that there was no obvious change between the long-term expression patterns of the 9 modules and the expression patterns in the ONHs 21 d post-ONC (Figures 4, 6B). These results indicated that the expression levels of the core genes in each module in the ONHs 90 d post-ONC were similar to those in the ONHs 21 d post-ONC, although the number of DEGs 90 d post-ONC was significantly decreased. In addition, the GO enrichment (biological process) analyses for the 90 d DEGs showed that axonogenesis-, neurogenesis- and nervous system development-related biological processes were significantly enriched in the downregulated DEGs (Figure 6C) and that cell proliferation-, response to external stimulus-, and cardiovascular system development-related biological processes were significantly enriched in the upregulated DEGs (Figure 6D).

### The Time Course of Changes in Glial Molecular Markers

**Figure 7:**
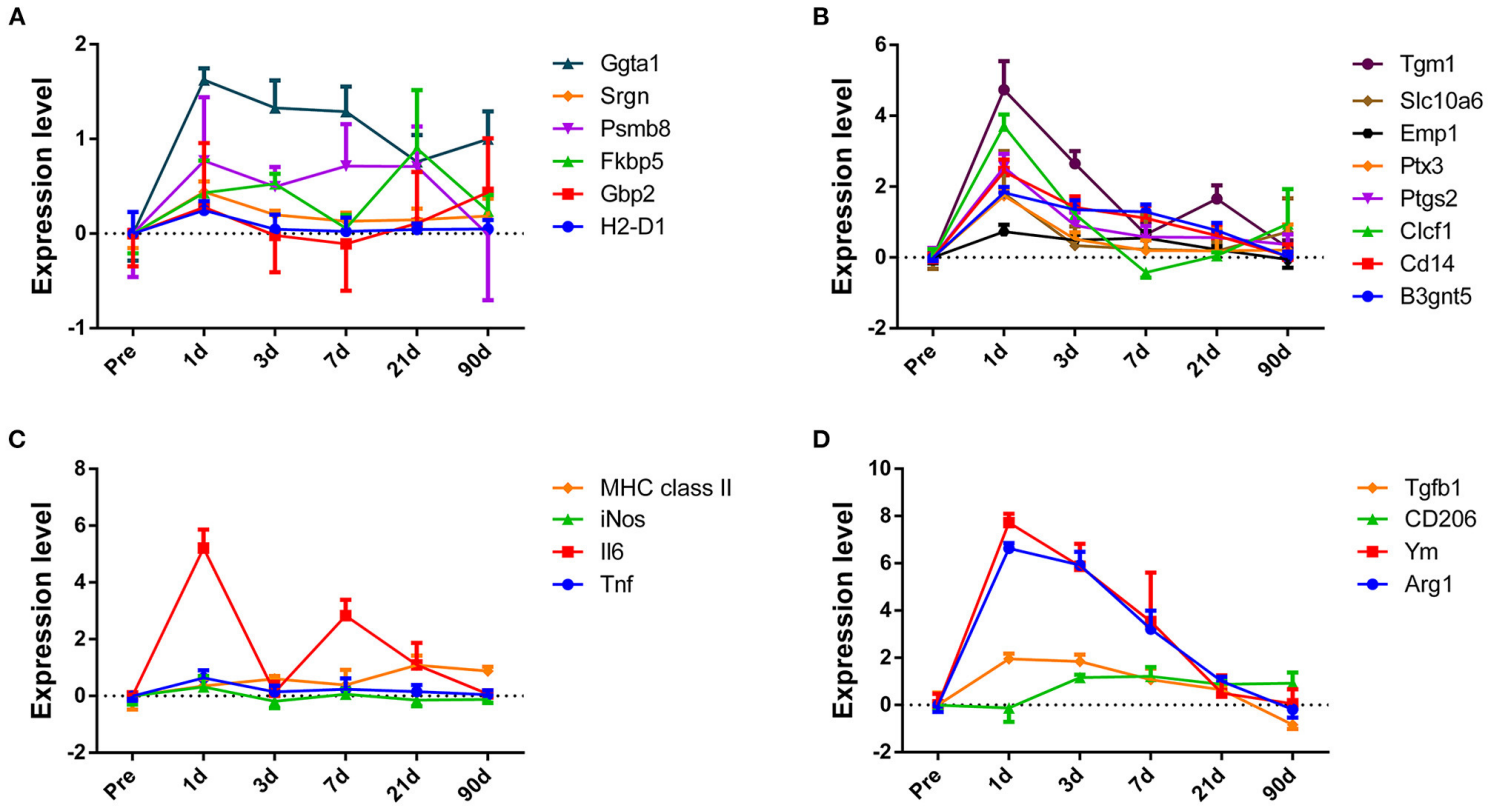
The changes in expression levels of subpopulations of astrocyte and microglial marker genes after crush injury. Overall, there was an obvious expression pattern difference between the subpopulation of astrocyte and microglial markers. **(A,B)** Genes expressed by A1- and A2-reactive astrocytes. **(C,D)** Genes expressed by M1 and M2 microglia, respectively.

After we obtained a big picture of the gene expression profile in ONH, we further tried to determine the specific expression pattern of glial cells, which accounted for the majority of cells in the ONH. Due to the heterogeneity of glial cells, we did not adopt markers that are commonly used. Instead, we selected markers that can represent subpopulations of astrocytes, microglia or oligodendrocytes. According to Liddelow's research (Liddelow et al., 2017), reactive astrocytes can be divided into the A1 and A2 types. In the A1 group, we found that A1 markers, such as H2-D1, Gbp2, Psmb8, Fkbp5, and Srgn, showed slightly upregulated expression at 1 dpc, and then, the expression dropped to baseline. Although a slight decline was observed in Ggta1 expression, it was still maintained at a high level at 90 dpc (Figure 7A). While the Fbln5 and Serping1 expression levels shared the same trend, both showed downregulated expression and then increased expression to baseline (Supplementary Figure 7A). The trend in the A2 group was more uniform, except Sphk1 expression, which sharply increased at 1 dpc and dropped to baseline over time. Specifically, the B3gnt5 and Emp1 levels slowly declined to baseline (Supplementary Figure 7B), while the Cd109, Cd14, Clcf1, Ptgs2, Ptx3, S100a10, Slc10a6, Tgm1, and Tm4sf1 levels rapidly dropped to baseline at 3 dpc or 7 dpc (Figure 7B). Additionally, we selected several genes that can represent two polarization states of microglia. Compared to those in the M2 group, the genes in the M1 group (Tnf, iNOS, MHC class II, Il6) changed more transiently. In the period of 1–7 days, the expression of most genes experienced a dramatic increase and then a sharp decline (Figure 7C). In the same period, the expression of the genes in the M2 group (Arg1, Ym1, CD206, and Tgf-β1) was still at a high level (Figure 7D). Oligodendrocytes and their precursors are in a resting state in the normal optic nerve; once injured, they adjust gene expression to express stage-dependent antigens to form the myelin sheaf (Ono et al., 2001; Barateiro and Fernandes, 2014). Although the expression levels of genes in different stages varied in degree, they showed a continuous downward trend and were maintained at a low level (data not shown).

## Discussion

Glial cells, including astrocytes, microglia and oligodendrocytes, play an important role in the optic nerve head, including providing support and nutrition as well as protection to axons. Recent studies have revealed that these cells are also responsible for waste clearance from the retina (Wang et al., 2020). In addition to functional diversity, glial cells show structural complexity in that they not only connect with axons but also interact with each other (Adams and Gallo, 2018; Jha et al., 2019). These findings indicate that glial cells may function through different mechanisms at the corresponding period. Injury to the optic nerve will disrupt this harmonious relationship, leading to a series of changes. However, most previous studies focused on changes caused by a certain molecule at a particular time point, which was limited and ignored many details. Now, by means of bioinformatics, without having to isolate a particular type of cell, we can obtain a more comprehensive and objective view of gene expression *in vivo*.

In this study, we adopted a novel analytical approach to analyze the data from the optic nerve head at different times after injury. PCA is a sophisticated method widely used for reducing the dimensions of multivariate problems and evaluating independence without losing much information (Liu et al., 2015). Based on the PCA results, we found that genes in the ONC group were divided into four separate clusters according to the time past injury. The injury-induced gene profile changed dramatically at 1 dpc, and then, the trend flattened to a resting level at 21 dpc. In addition, sample clustering showed that the time-dependent changes can be divided into two distinct phases, demonstrating a different expression profile between the early and late stages of optic nerve crush. To further reveal the pattern of change, we identified differentially expressed genes (DEGs). The number of DEGs gradually decreased over time and shared the same trend as the PCA results, verifying the observation above.

WGCNA, a newly developed bioinformatics technique, allows dissection of various pathological events in an injured optic nerve via transcriptome analyses (Langfelder and Horvath, 2008). Briefly, the transcriptome not only reflects the identity of the cell but also represents the physiological/pathological state it experienced. Thus, within the injured optic nerve at different times post-injury, various pathological cellular events could be reflected by transcriptional programs of the injured optic nerve. Genes with similar expression patterns across all samples will innately cluster together. These clustered cores, often called modules, represent genes involved in the execution of particular biological functions. Moreover, gene functions in particular cell types sometimes also cluster together, and thus, some modules could be specific for certain cells. These events actually represent cell type-specific functions.

From the mapping of WGCNA, we found that different pathological processes occurred in different orders and degrees with the progression of injury. From the results, we can obtain a more holistic view of pathological injury processes at a systemic level.

Genes related to neuronal differentiation, axonogenesis and neurogenesis in M3, M4, and M6 showed persistently downregulated expression, which may suggest a decline in regenerative capacity. These modules may reflect the pathological state of injured axons in the optic nerve. Conversely, genes related to inflammation, immune response and microglial activation showed upregulated expression in M2 and M5, suggesting ongoing repair progress that led to an adverse microenvironment for axonal regeneration. Correspondingly, these modules may reflect the pathological state of glial cells or immune cells around the injured site. Moreover, we performed hub gene network analyses based on a pairwise Pearson correlation matrix, aiming to identify genes that could potentially serve as therapeutic targets to alter pathological progression. For example, Tyrobp, also known as DAP12, is one of the hub genes in M2. This molecule is expressed in microglia and forms a receptor complex with TREM2. A study showed that deficiency of the complex led to improved pathological and functional outcomes after TBI in mice (Saber et al., 2017). In M4, Mapt was reported to regulate axon initiation (Sohn et al., 2019). Taken together, the results provide a broader and more comprehensive view of pathological progress in ONC.

Glial cells form a complex and delicate network to monitor subtle changes and respond correspondingly to maintain the optic nerve. Astrocytes and microglia are populations of heterogeneous cells, and they change their phenotype and function depending on their environment or the stimulation they receive (Adams and Gallo, 2018; Yazdankhah et al., 2021). Thus, during the different periods of injury, these cells present different states in response to the situation. In this paper, we selected several genes that can represent different states of astrocytes, microglial cells and oligodendrocytes to observe their changes over time.

The Barres laboratory characterized two types of reactive astrocytes named A1 and A2. These researchers found that A1 astrocytes secrete a neurotoxin that promotes neuronal and oligodendrocyte cell death. In contrast, A2 astrocytes promote neuronal survival and growth (Liddelow et al., 2017). In the optic nerve, eight genes of the A1 astrocyte phenotype were detected, and most genes changed mildly after injury. Reactive astrocytes in the optic nerve expressed all 12 genes used to identify A2 astrocytes. These genes showed dramatically upregulated expression at 1 dpc, and the expression slowly descended to a resting level until 21 dpc. Reactive astrocytes preferentially display an A2-like phenotype at least until 1 dpc. This result may coincide with Anderson's finding that astrocytes may aid axon regeneration at an early stage (Anderson et al., 2016). In the Barres study, the researchers examined the changes in astrocytes in the retina and drew a same conclusion made in brain. In this research, we analyzed changes in astrocytes in the optic nerve. The phenotypes between these types of cells do not match perfectly, indicating that astrocytes in the retina and optic nerve head may be two distinct populations and respond differently to injury. The reason for this difference may be the heterogeneous astrocytes. Astrocytes display distinct regional identities and functional properties (Tsai et al., 2012; John Lin et al., 2017), and even the severity of the insult or the variety of CNS injuries could result in widely varied cellular, molecular and structural changes (Zamanian et al., 2012).

Normal tissue healing, such as skin and muscle healing, proceeds through three phases. Phase I (within hours, peaking at 1–3 days post-injury) is characterized by an initial inflammatory response; Phase II (spans from 2 to 10 days) is characterized by tissue formation, such as angiogenesis and extracellular matrix formation; and Phase III (usually begins a week post-injury and can last for months) is characterized by tissue remodeling and inflammatory resolution. These three phases proceed in an orderly manner to ensure organized tissue healing, which relies heavily on the proper function of microglia or macrophages. In the CNS, similar phases also existed after injury, but the gap between them was blurred, and the coordination was disrupted, resulting in a secondary injury to axons. The results showed that both M1 and M2 markers had upregulated expression at 1 dpc, which may indicate that a mixed response occurred. Then, most M1 markers dropped rapidly to baseline at 3 dpc, while M2 markers remained at a high expression level and decreased slowly. An M1 to M2 shift was observed during this period; however, we cannot exclude the possibility that M2 microglia migrate from the periphery to the injury site. A reasonable explanation may be that in the early phase, activation of M1 recruits immune cells and reactive astrocytes to restrict the lesion, while M2 microglial responses dominate the late phase and might contribute to phagocytosis of cell debris. However, we cannot make a definitive separation between the two states of microglia. Notably, classic identification of macrophages or microglia based on M1 (proinflammatory) and M2 (anti-inflammatory) polarization paradigms cannot be used for further studies (Hu et al., 2015; Lan et al., 2017). An increasing number of studies have shown that these populations represent a continuous spectrum of phenotypes, with overlapping characteristics and markers (Murray et al., 2014).

Oligodendrocytes are another important component of glial cells in the CNS. Remyelination is necessary for the re-establishment of axonal conduction velocity and metabolic support to the axons. Here, we selected several genes that represent different stages of oligodendrocyte differentiation. Generally, the overall expression profile showed a downward trend over time, and expression was maintained at a low level. In the healthy CNS, myelin is produced throughout life, and spontaneous remyelination occurs readily in response to insults. However, remyelination eventually fails after ONC. One acceptable explanation is that the glial scar prevents the migration of NG2 cells to the proximal and distal ends of the lesion. Another view suggests that surviving mature oligodendrocytes re-extend their processes to contact axons rather than proliferation. In addition, several studies have described beneficial and detrimental roles played by astrocytes in remyelination. The failure of remyelination may be related to the state of astrocytes. Exactly which astrocytes have an effect on remyelination is unknown. The connection between oligodendrocytes and astrocytes or microglia needs to be further studied.

In our research, we described a systematic and comprehensive analysis of the gene expression profile in the optic nerve head mainly related to glial cells after optic nerve crush. With the progress of pathology, we found that different glial cells represent corresponding changes. Currently, some preclinical drugs target microglial polarization or astrocyte activation. Thus, a better understanding of the time-dependent reorganization of glial cells may be necessary to maximize the potential of combinatorial approaches. On a macro level, we observed the pathological process in the injured optic nerve. Another concern is further classifying pathologies according to glial cellular profiles. With single-cell high-throughput sequencing, this goal is being achieved.

In conclusion, we adopted a novel bioinformatics tool to analyze the transcriptome data of the ONH after optic nerve crush. First, the PCA results showed different expression patterns of the ONH at the early and late stages. Second, WGCNA described a comprehensive pattern of molecular pathological progress in the ONH after optic nerve crush. Based on this, we further explored the hub genes that may be potential therapeutic targets. Finally, we observed gliosis in the ONH and revealed the different responses of subpopulations of astrocytes and microglia according to related markers.

## Data Availability Statement

The datasets presented in this study can be found in online repositories. The names of the repository/repositories and accession number(s) can be found in the article/Supplementary Material.

## Author Contributions

Y-BP designed the study and wrote the article. Y-BP, YS, H-JL, L-YZ, JZ, and D-FF analyzed and interpreted the data. All authors reviewed and approved the final manuscript.

## Funding

This work was funded by the National Natural Science Foundation of China (Grant Nos. 81772059, 82071287, and 81870916), and the MOE Frontier Science Center for Brain Science & Brain-Machine Integration, Zhejiang University.

## Conflict of Interest

The authors declare that the research was conducted in the absence of any commercial or financial relationships that could be construed as a potential conflict of interest.

## Publisher's Note

All claims expressed in this article are solely those of the authors and do not necessarily represent those of their affiliated organizations, or those of the publisher, the editors and the reviewers. Any product that may be evaluated in this article, or claim that may be made by its manufacturer, is not guaranteed or endorsed by the publisher.

## Supplementary Material

Supplementary Figure S1 PCA and hierarchical clustering of the complete dataset. **(A)** Two-dimensional PCA of the complete dataset (55 ONHs). Uninjured control ONHs were labeled as rounds. The injured ONHs were labeled as triangles. **(B)** Three-dimensional PCA of the complete dataset. The time points following ONC were labeled with different colors. **(C)** Hierarchical clustering of the complete dataset based on all gene profiles. PCA, principle component analysis; ONC, optic nerve crush; ONH, optic nerve head.

Supplementary Figure S2 Hierarchical clustering of the 45 ONHs based on all gene profiles. ONH, optic nerve head.

Supplementary Figure S3 Differentially expression analyses of injured ONHs 1/3/7/21d post-ONC. **(A)** Based on different threshold conditions (logFC), the numbers of DEGs at each time point of ONC were shown. **(B–E)** Volcano plot of DEGs in ONHs 1/3/7/21d post-ONC compared with uninjured control ONHs. Red points represent DEGs. DEG, differentially expressed gene; ONC, optic nerve crush; ONH, optic nerve head.

Supplementary Figure S4 Cell type enrichment analyses for M1-M5 modules. **(A–E)** Cell type enrichment analyses for M1-M5 modules.

Supplementary Figure S5 Cell type enrichment analyses for M6-M9 modules. **(A–D)** Cell type enrichment analyses for M6-M9 modules.

Supplementary Figure S6 Visualized network of each module. **(A–D)** Visualized networks based on top 250 gene connections of M1 **(A)**, M3 **(B)**, M7 **(C)**, M9 **(D)**, and M8 **(E)** modules. The top 5 hub genes based on degree in each module were shown.

Supplementary Figure S7 Other markers expressed by A1 and A2 reactive astrocyte.

Supplementary Table S1 GO terms of each module.

Supplementary Table S2 Cell type terms of each module.

Supplementary Table S3 DEGs at each time point after ONC.

## Abbreviations

ONC: Optic nerve crush
ONH: optic nerve head
WGCNA: weighted gene coexpression network analysis
CNS: central nervous system
RMA: robust multi-array average
KNN: k-Nearest Neighbor
PCA: Principle component analysis
DEA: Differentially expression analysis
DEGs: differentially expressed genes
TOM: topological overlap matrix
ME: module eigengene
MM: module membership
GO: Gene Ontology.

## References

[B1] Adams K. L. Gallo V. (2018). The diversity and disparity of the glial scar. Nat. Neurosci. 21, 9–15. 10.1038/s41593-017-0033-9 29269757 PMC5937232

[B2] Agudo M. Perez-Marin M. C. Lonngren U. Sobrado P. Conesa A. Canovas I.. (2008). Time course prodoi filing of the retinal transcriptome after optic nerve transection and optic nerve crush. Mol. Vis. 14, 1050–1063. 18552980 PMC2426719

[B3] Anderson M. A. Burda J. E. Ren Y. Ao Y. O'shea T. M. Kawaguchi R.. (2016). Astrocyte scar formation aids central nervous system axon regeneration. Nature 532, 195–200. 10.1038/nature17623 27027288 PMC5243141

[B4] Barateiro A. Fernandes A. (2014). Temporal oligodendrocyte lineage progression: in vitro models of proliferation, differentiation and myelination. Biochim. Biophys. Acta 1843, 1917–1929. 10.1016/j.bbamcr.2014.04.018 24768715

[B5] Bei F. Lee H. H. C. Liu X. Gunner G. Jin H. Ma L.. (2016). Restoration of visual function by enhancing conduction in regenerated axons. Cell 164, 219–232. 10.1016/j.cell.2015.11.036 26771493 PMC4863988

[B6] Garcia-Laencina P. J. Abreu P. H. Abreu M. H. Afonoso N. (2015). Missing data imputation on the 5-year survival prediction of breast cancer patients with unknown discrete values. Comput. Biol. Med. 59, 125–133. 10.1016/j.compbiomed.2015.02.006 25725446

[B7] Hilla A. M. Diekmann H. Fischer D. (2017). Microglia are irrelevant for neuronal degeneration and axon regeneration after acute injury. J. Neurosci. 37, 6113–6124. 10.1523/JNEUROSCI.0584-17.2017 28539419 PMC6596505

[B8] Hu X. Leak R. K. Shi Y. Suenaga J. Gao Y. Zheng P.. (2015). Microglial and macrophage polarization-new prospects for brain repair. Nat. Rev. Neurol. 11, 56–64. 10.1038/nrneurol.2014.207 25385337 PMC4395497

[B9] Huang Da W. Sherman B. T. Lempicki R. A. (2009). Systematic and integrative analysis of large gene lists using DAVID bioinformatics resources. Nat. Protoc. 4, 44–57. 10.1038/nprot.2008.211 19131956

[B10] Irizarry R. A. Hobbs B. Collin F. Beazer-Barclay Y. D. Antonellis K. J. Scherf U.. (2003). Exploration, normalization, and summaries of high density oligonucleotide array probe level data. Biostatistics 4, 249–264. 10.1093/biostatistics/4.2.249 12925520

[B11] Jha M. K. Jo M. Kim J. H. Suk K. (2019). Microglia-astrocyte crosstalk: an intimate molecular conversation. Neuroscientist 25, 227–240. 10.1177/1073858418783959 29931997

[B12] John Lin C. C. Yu K. Hatcher A. Huang T. W. Lee H. K. Carlson J.. (2017). Identification of diverse astrocyte populations and their malignant analogs. Nat. Neurosci. 20, 396–405. 10.1038/nn.4493 28166219 PMC5824716

[B13] Lan X. Han X. Li Q. Yang Q. W. Wang J. (2017). Modulators of microglial activation and polarization after intracerebral haemorrhage. Nat. Rev. Neurol. 13, 420–433. 10.1038/nrneurol.2017.69 28524175 PMC5575938

[B14] Langfelder P. Horvath S. (2008). WGCNA: an R package for weighted correlation network analysis. BMC Bioinformatics 9:559. 10.1186/1471-2105-9-559 19114008 PMC2631488

[B15] Li Y. He X. Kawaguchi R. Zhang Y. Wang Q. Monavarfeshani A.. (2020). Microglia-organized scar-free spinal cord repair in neonatal mice. Nature 587, 613–618. 10.1038/s41586-020-2795-6 33029008 PMC7704837

[B16] Liddelow S. A. Guttenplan K. A. Clarke L. E. Bennett F. C. Bohlen C. J. Schirmer L.. (2017). Neurotoxic reactive astrocytes are induced by activated microglia. Nature 541, 481–487. 10.1038/nature21029 28099414 PMC5404890

[B17] Liu R. S. Jin G. H. Xiao D. R. Li H. M. Bai F. W. Tang Y. J. (2015). Screening of the key volatile organic compounds of Tuber melanosporum fermentation by aroma sensory evaluation combination with principle component analysis. Sci. Rep. 5:17954. 10.1038/srep17954 26655663 PMC4675963

[B18] Moore D. L. Blackmore M. G. Hu Y. Kaestner K. H. Bixby J. L. Lemmon V. P.. (2009). KLF family members regulate intrinsic axon regeneration ability. Science 326, 298–301. 10.1126/science.1175737 19815778 PMC2882032

[B19] Murray P. J. Allen J. E. Biswas S. K. Fisher E. A. Gilroy D. W. Goerdt S.. (2014). Macrophage activation and polarization: nomenclature and experimental guidelines. Immunity 41, 14–20. 10.1016/j.immuni.2014.06.008 25035950 PMC4123412

[B20] Ono K. Kagawa T. Tsumori T. Yokota S. Yasui Y. (2001). Morphological changes and cellular dynamics of oligodendrocyte lineage cells in the developing vertebrate central nervous system. Dev. Neurosci. 23, 346–355. 10.1159/000048718 11756750

[B21] Pan Y. B. Wang S. Yang B. Jiang Z. Lenahan C. Wang J.. (2020). Transcriptome analyses reveal molecular mechanisms underlying phenotypic differences among transcriptional subtypes of glioblastoma. J. Cell. Mol. Med. 24, 3901–3916. 10.1111/jcmm.14976 32091665 PMC7171397

[B22] Park K. K. Liu K. Hu Y. Smith P. D. Wang C. Cai B.. (2008). Promoting axon regeneration in the adult CNS by modulation of the PTEN/mTOR pathway. Science 322, 963–966. 10.1126/science.1161566 18988856 PMC2652400

[B23] Pernet V. Joly S. Jordi N. Dalkara D. Guzik-Kornacka A. Flannery J. G.. (2013). Misguidance and modulation of axonal regeneration by Stat3 and Rho/ROCK signaling in the transparent optic nerve. Cell Death Dis. 4:e734. 10.1038/cddis.2013.266 23868067 PMC3730436

[B24] Qu J. Jakobs T. C. (2013). The time course of gene expression during reactive gliosis in the optic nerve. PLoS ONE 8:e67094. 10.1371/journal.pone.0067094 23826199 PMC3694957

[B25] Ritchie M. E. Phipson B. Wu D. Hu Y. Law C. W. Shi W.. (2015). limma powers differential expression analyses for RNA-sequencing and microarray studies. Nucleic Acids Res. 43:e47. 10.1093/nar/gkv007 25605792 PMC4402510

[B26] Saber M. Kokiko-Cochran O. Puntambekar S. S. Lathia J. D. Lamb B. T. (2017). Triggering receptor expressed on myeloid cells 2 deficiency alters acute macrophage distribution and improves recovery after traumatic brain injury. J. Neurotrauma 34, 423–435. 10.1089/neu.2016.4401 26976047

[B27] Sharma T. P. Mcdowell C. M. Liu Y. Wagner A. H. Thole D. Faga B. P.. (2014). Optic nerve crush induces spatial and temporal gene expression patterns in retina and optic nerve of BALB/cJ mice. Mol. Neurodegener. 9:14. 10.1186/1750-1326-9-14 24767545 PMC4113182

[B28] Shoja M. M. Oyesiku N. M. (2014). Clinical anatomy of the cranial nerves. Clin. Anat. 27, 2–3. 10.1002/ca.22365 24343774

[B29] Sohn P. D. Huang C. T. Yan R. Fan L. Tracy T. E. Camargo C. M.. (2019). Pathogenic tau impairs axon initial segment plasticity and excitability homeostasis. Neuron 104, 458–470.e455. 10.1016/j.neuron.2019.08.008 31542321 PMC6880876

[B30] Sun F. Park K. K. Belin S. Wang D. Lu T. Chen G.. (2011). Sustained axon regeneration induced by co-deletion of PTEN and SOCS3. Nature 480, 372–375. 10.1038/nature10594 22056987 PMC3240702

[B31] Tedeschi A. Bradke F. (2017). Spatial and temporal arrangement of neuronal intrinsic and extrinsic mechanisms controlling axon regeneration. Curr. Opin. Neurobiol. 42, 118–127. 10.1016/j.conb.2016.12.005 28039763

[B32] Tsai H. H. Li H. Fuentealba L. C. Molofsky A. V. Taveira-Marques R. Zhuang H.. (2012). Regional astrocyte allocation regulates CNS synaptogenesis and repair. Science 337, 358–362. 10.1126/science.1222381 22745251 PMC4059181

[B33] Wang X. Lou N. Eberhardt A. Yang Y. Kusk P. Xu Q.. (2020). An ocular glymphatic clearance system removes β-amyloid from the rodent eye. Sci. Transl. Med. 12:eaaw3210. 10.1126/scitranslmed.aaw3210 32213628 PMC7356596

[B34] Williams P. R. Benowitz L. I. Goldberg J. L. He Z. (2020). Axon regeneration in the mammalian optic nerve. Annu. Rev. Vis. Sci. 6, 195–213. 10.1146/annurev-vision-022720-094953 32936739

[B35] Yazdankhah M. Shang P. Ghosh S. Hose S. Liu H. Weiss J.. (2021). Role of glia in optic nerve. Prog. Retin. Eye Res. 81:100886. 10.1016/j.preteyeres.2020.100886 32771538 PMC7865017

[B36] Zamanian J. L. Xu L. Foo L. C. Nouri N. Zhou L. Giffard R. G.. (2012). Genomic analysis of reactive astrogliosis. J. Neurosci. 32, 6391–6410. 10.1523/JNEUROSCI.6221-11.2012 22553043 PMC3480225

[B37] Zhang B. Horvath S. (2005). A general framework for weighted gene co-expression network analysis. Stat. Appl. Genet. Mol. Biol. 4:17. 10.2202/1544-6115.1128 16646834

