# Supplementary figures and images for: Transcriptome Analyses Reveal Systematic Molecular Pathology After Optic Nerve Crush
Source: Front Cell Neurosci. 2022 Jan 10;15:800154. doi: 10.3389/fncel.2021.800154 (PMC8784559; doi:10.3389/fncel.2021.800154)

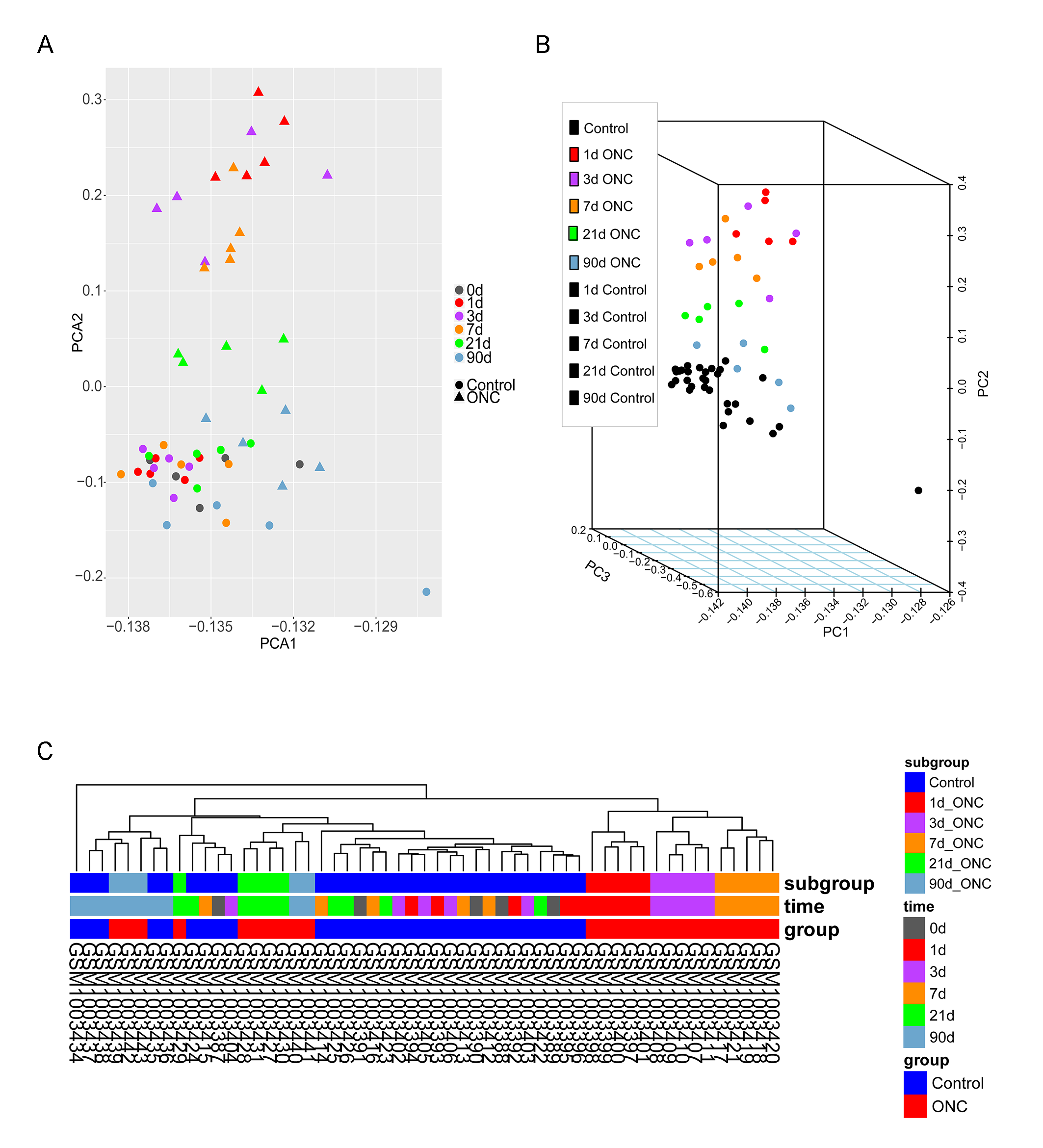

Supplement: Supplementary Figure S1 — PCA and hierarchical clustering of the complete dataset. (A) Two-dimensional PCA of the complete dataset (55 ONHs). Uninjured control ONHs were labeled as rounds. The injured ONHs were labeled as triangles. (B) Three-dimensional PCA of the complete dataset. The time points following ONC were labeled with different colors. (C) Hierarchical clustering of the complete dataset based on all gene profiles. PCA, principle component analysis; ONC, optic nerve crush; ONH, optic nerve head. [file Image_1.TIF]

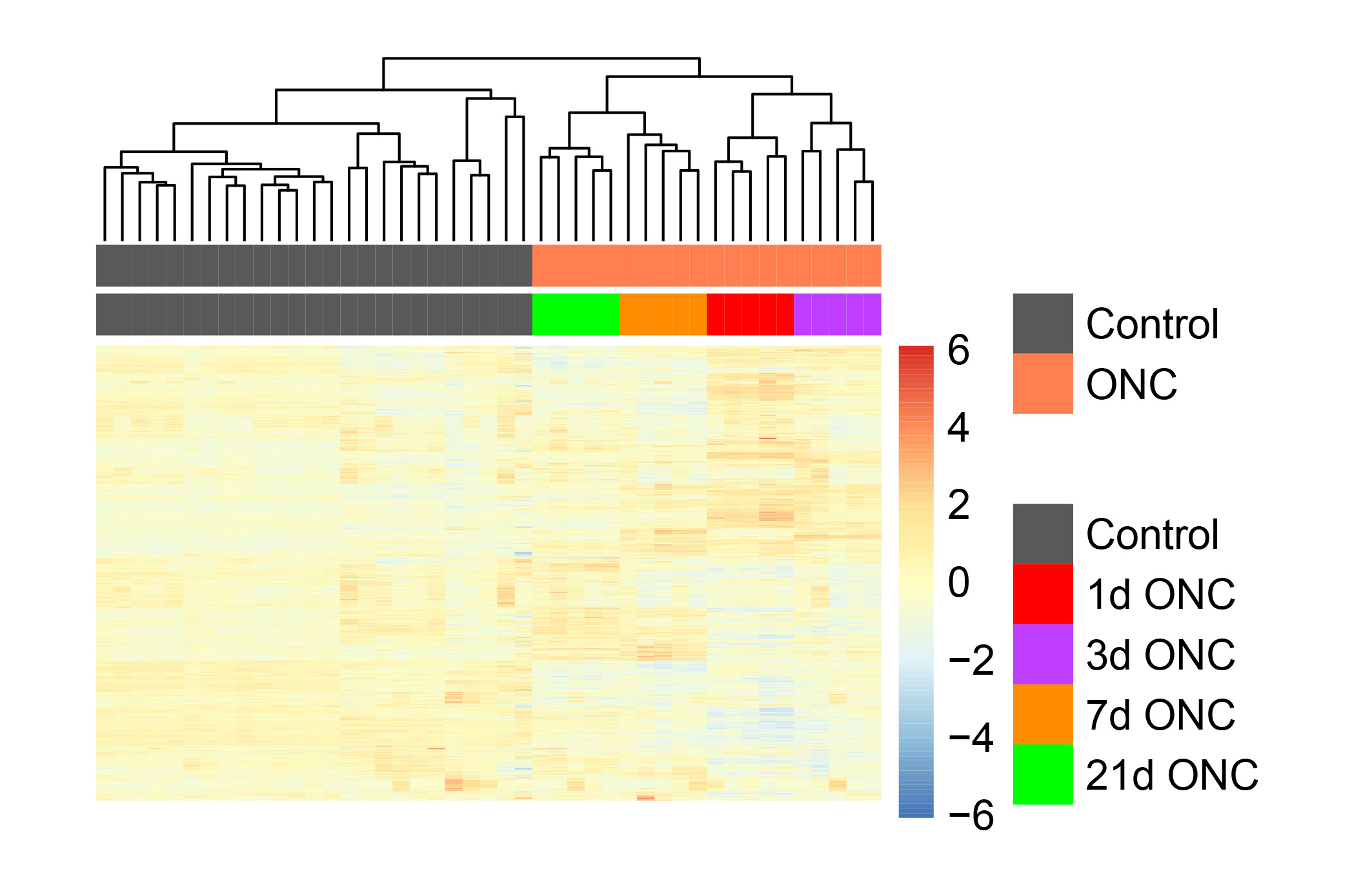

Supplement: Supplementary Figure S2 — Hierarchical clustering of the 45 ONHs based on all gene profiles. ONH, optic nerve head. [file Image_2.JPEG]

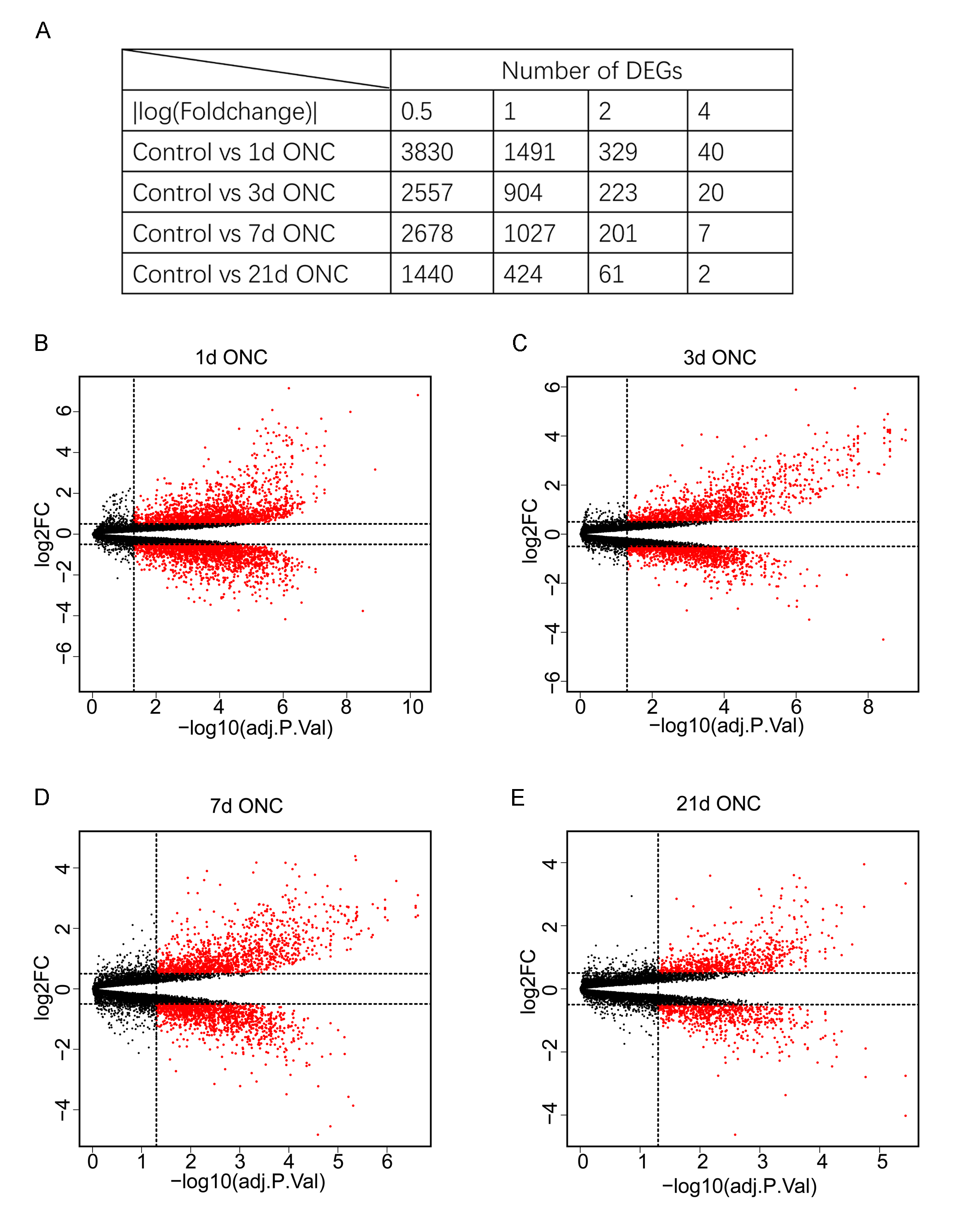

Supplement: Supplementary Figure S3 — Differentially expression analyses of injured ONHs 1/3/7/21d post-ONC. (A) Based on different threshold conditions (logFC), the numbers of DEGs at each time point of ONC were shown. (B–E) Volcano plot of DEGs in ONHs 1/3/7/21d post-ONC compared with uninjured control ONHs. Red points represent DEGs. DEG, differentially expressed gene; ONC, optic nerve crush; ONH, optic nerve head. [file Image_3.TIF]

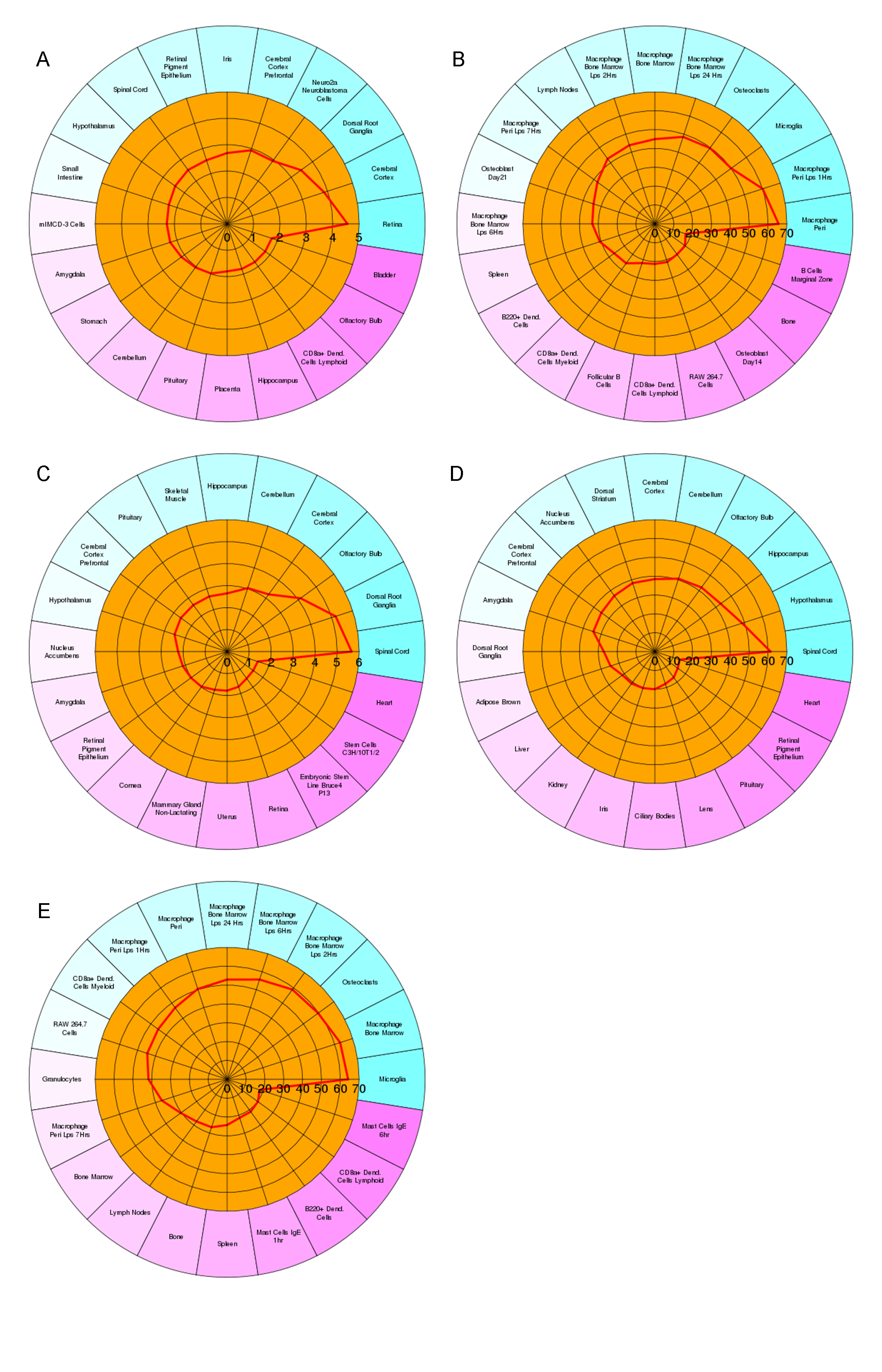

Supplement: Supplementary Figure S4 — Cell type enrichment analyses for M1-M5 modules. (A–E) Cell type enrichment analyses for M1-M5 modules. [file Image_4.TIF]

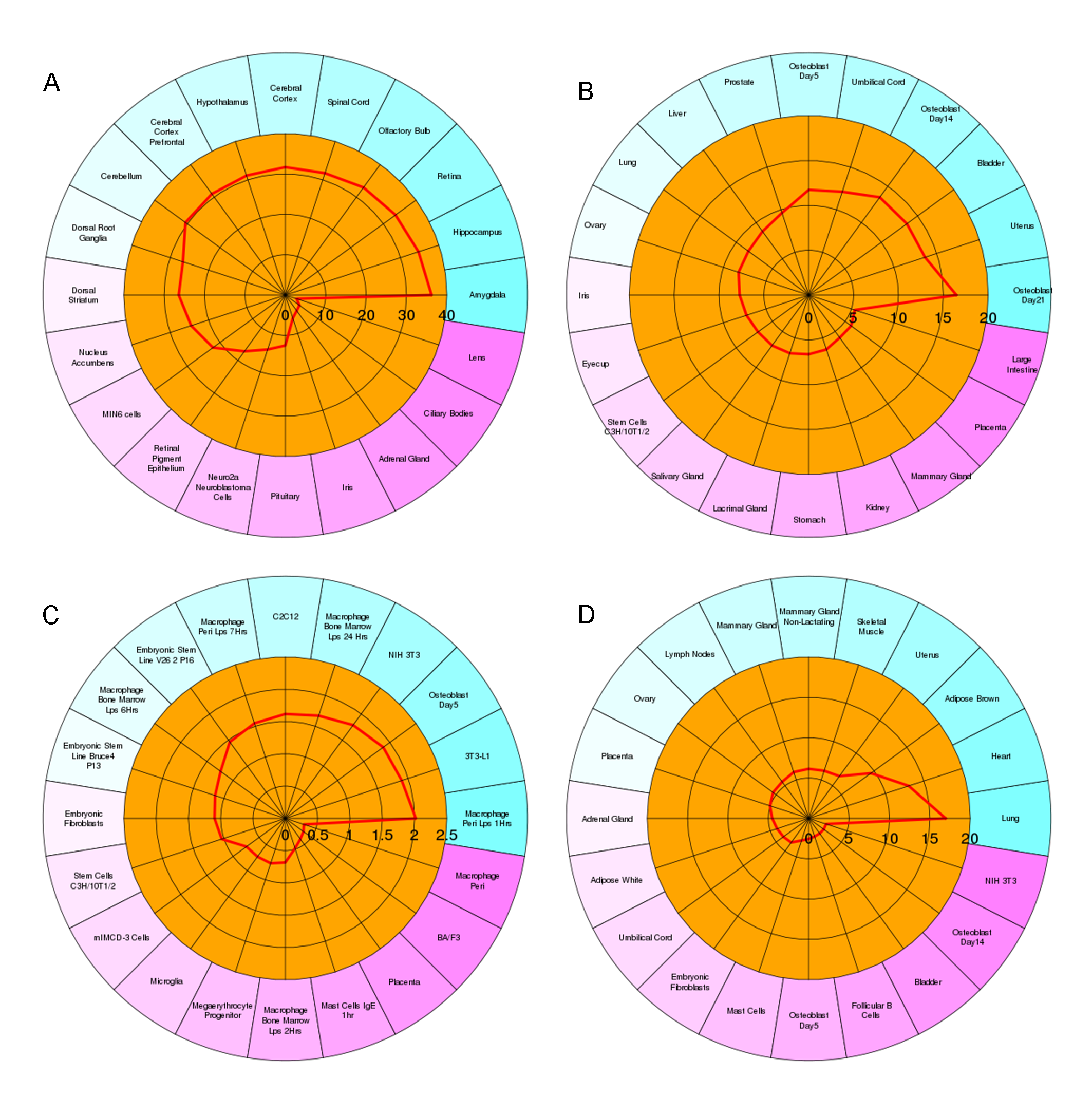

Supplement: Supplementary Figure S5 — Cell type enrichment analyses for M6-M9 modules. (A–D) Cell type enrichment analyses for M6-M9 modules. [file Image_5.TIF]

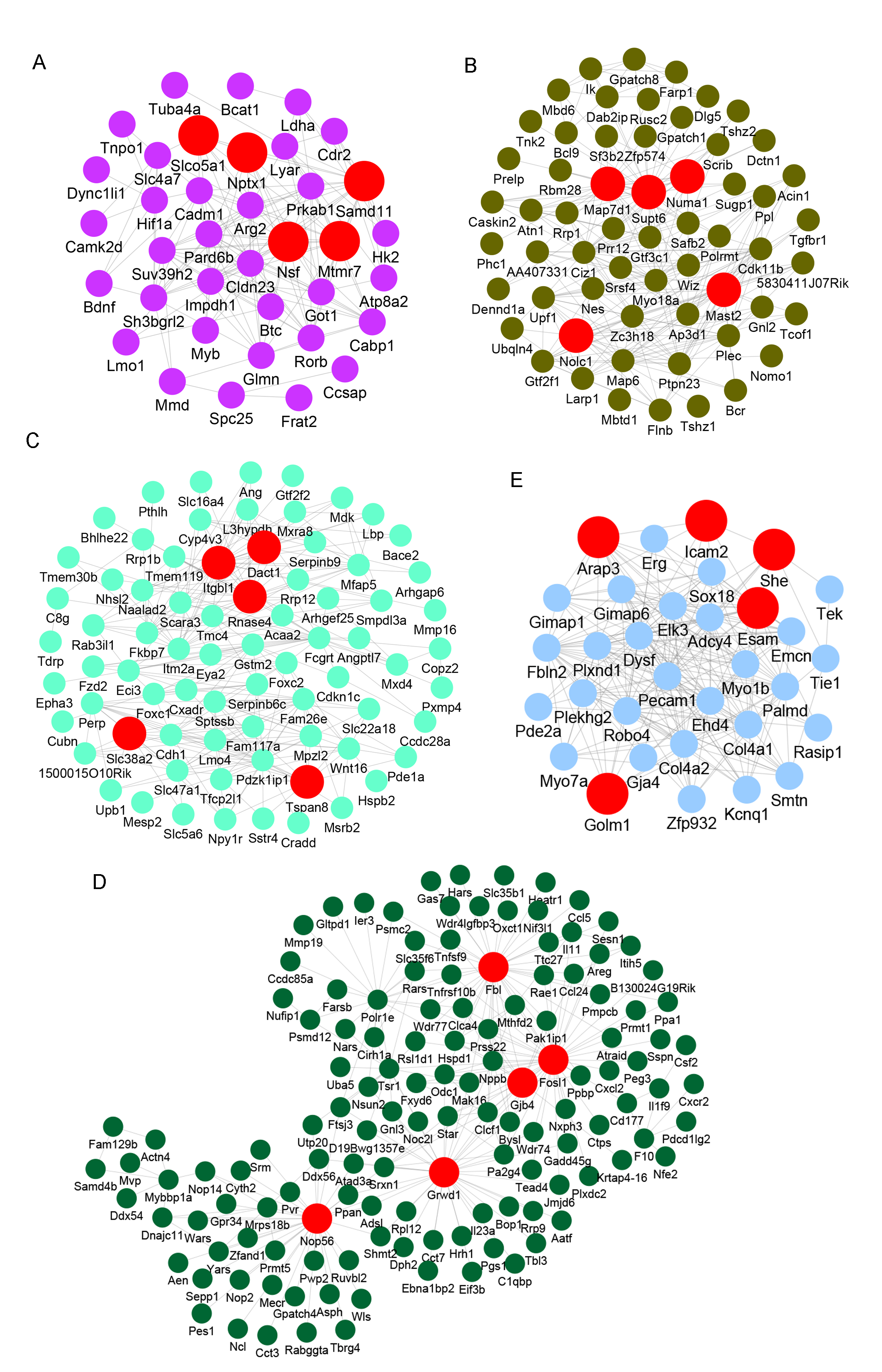

Supplement: Supplementary Figure S6 — Visualized network of each module. (A–D) Visualized networks based on top 250 gene connections of M1 (A), M3 (B), M7 (C), M9 (D), and M8 (E) modules. The top 5 hub genes based on degree in each module were shown. [file Image_6.TIF]

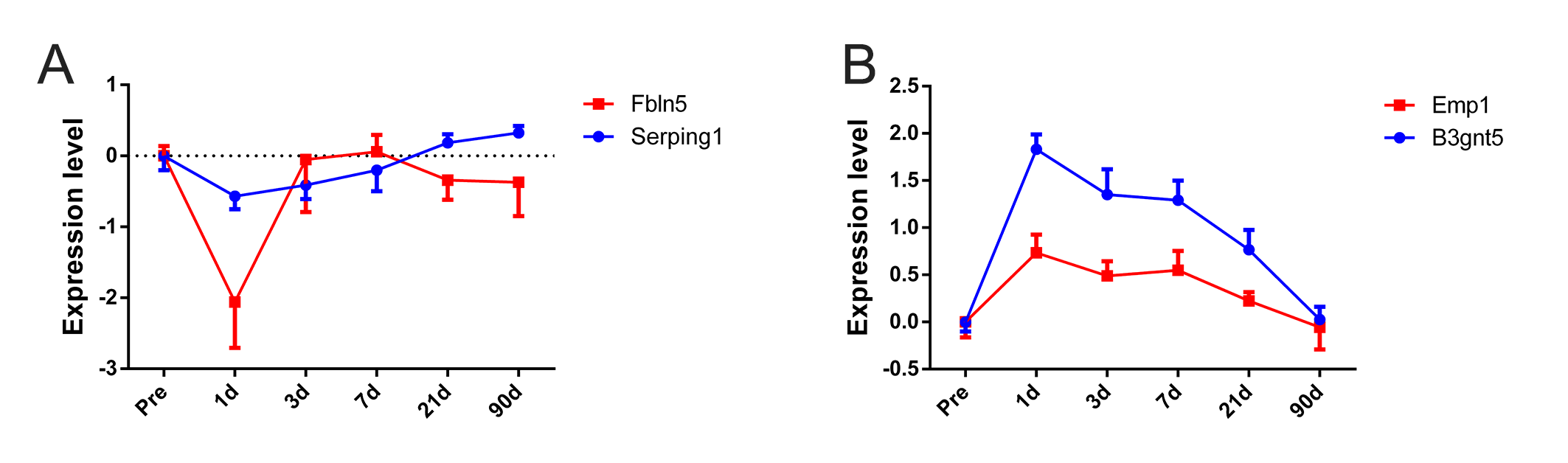

Supplement: Supplementary Figure S7 — Other markers expressed by A1 and A2 reactive astrocyte. [file Image_7.TIF]
